# Peripheral Membrane Proteins: Promising Therapeutic Targets across Domains of Life

**DOI:** 10.3390/membranes11050346

**Published:** 2021-05-08

**Authors:** Deborah M. Boes, Albert Godoy-Hernandez, Duncan G. G. McMillan

**Affiliations:** 1 Department of Biotechnology, Delft University of Technology, Van der Maasweg 9, NL-2629 HZ Delft, The Netherlands; D.M.Boes@student.tudelft.nl (D.M.B.); A.GodoyHernandez@tudelft.nl (A.G.-H.); 2 School of Fundamental Sciences, Massey University, Palmerston North, Private Bag 11 222, New Zealand

**Keywords:** peripheral membrane proteins, human diseases, GPI-anchored proteins, electrostatic interactions, hydrophobic membrane anchor, drug targets

## Abstract

Membrane proteins can be classified into two main categories—integral and peripheral membrane proteins—depending on the nature of their membrane interaction. Peripheral membrane proteins are highly unique amphipathic proteins that interact with the membrane indirectly, using electrostatic or hydrophobic interactions, or directly, using hydrophobic tails or GPI-anchors. The nature of this interaction not only influences the location of the protein in the cell, but also the function. In addition to their unique relationship with the cell membrane, peripheral membrane proteins often play a key role in the development of human diseases such as African sleeping sickness, cancer, and atherosclerosis. This review will discuss the membrane interaction and role of periplasmic nitrate reductase, CymA, cytochrome *c,* alkaline phosphatase, ecto-5’-nucleotidase, acetylcholinesterase, alternative oxidase, type-II NADH dehydrogenase, and dihydroorotate dehydrogenase in certain diseases. The study of these proteins will give new insights into their function and structure, and may ultimately lead to ground-breaking advances in the treatment of severe diseases.

## 1. Introduction

The lipid membrane surrounding cells and their compartments hosts proteins that perform functions essential for both cell physiology and disease progression. They are, by definition, located at the interface between two different environments, such as between the cytoplasm and the extracellular space, or between the mitochondrial matrix and the intermembrane space [1]. Membrane proteins constitute about a third of all human proteins and are categorized into two broad categories depending on their location [2]: integral membrane proteins (IMPs or ‘intrinsic proteins’) and peripheral membrane proteins (PMPs). 

**Figure 1:**
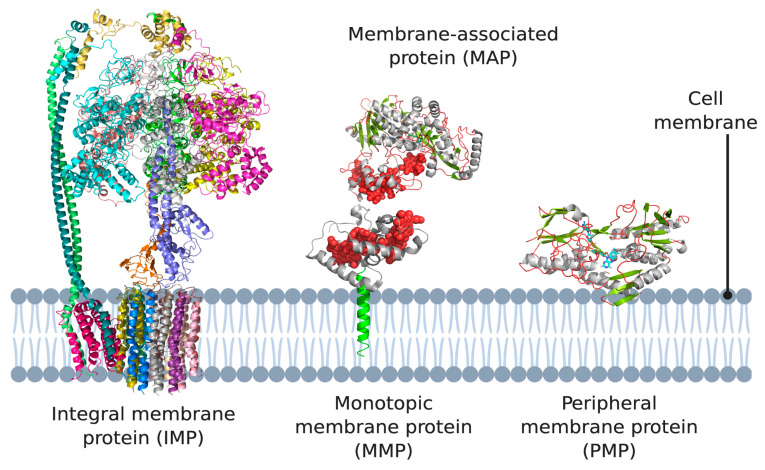
Different types of membrane proteins shown in the cell membrane. Integral membrane proteins are shown to span the entire membrane *(Escherichia coli* ATP synthase as example). Peripheral membrane proteins can be categorized into two subtypes: proteins that only associate with the membrane via electrostatic or hydrophobic interactions (*Caldalkalibacillus thermarum* type-II NADH dehydrogenase as example), and proteins that anchor themselves in the lipid bilayer using a hydrophobic segment that does not span the entire membrane (*Desulfovibrio vulgaris* NrfH as example). 3D structural cartoons of *E. coli* ATP synthase (PDB:5T4Q) [4], *D. vulgaris* NrfH (PDB:2J7A) [5], *Shewanella frigidimarina* flavocytochrome *c*_3_ (PDB:1Y0P) [6], and *C. thermarum* NDH-2 (PDB:6BDO) [7] were rendered with PyMol and the figure compiled in BioRender.com [8].

IMPs are embedded in the phospholipid bilayer (Figure 1) and contain one or more membrane-spanning domains (transmembrane proteins). An associated class of proteins are monotopic membrane proteins (MMPs; Figure 1) [3], which functionally interact with the environment on one side of the membrane and are akin to a lipid-anchored PMP. We class them as a subsection of ‘peripheral membrane proteins’ in the context of this review.

PMPs are a diverse group including proteins that associate directly with the lipid membrane and face one side of a lipid membrane (a true peripheral membrane protein; PMP, Figure 1) or indirectly via interactions with integral membrane proteins (a membrane-associated protein; MAP, Figure 1). The core focus of this review are PMPs, although MAPs directly associate with the aforementioned monotopic membrane proteins, referred to as subset of PMPs in the context of this review. PMPs associate with the membrane using a variety of different mechanisms. They can ‘nestle’ into the membrane, interacting via a hydrophobic patch with the membrane interior, or only associate via interactions with the polar head groups of the phospholipids [9]. Two subsets of peripheral membrane proteins which we particularly emphasize in this review are lipid-anchored proteins, which are anchored to the lipid bilayer by a special functionalized lipid embedded in the membrane, and quinone oxidoreductases, which play a key role in the electron transport chains of both oxidative phosphorylation and photosynthesis, as both pathways use quinones as their electron carrier. It should also be noted that association of some peripheral membrane proteins with the membrane is reversible, and some PMPs can be detached from the membrane by altering the pH or concentration, however, this is certainly not the case for monotopic membrane proteins and IMPs, which are permanently attached to the membrane [10,11,12].

PMPs also play a key role in metabolic pathways, making them attractive propositions in the search for cures for diseases ranging from tuberculosis [13] and cancer [14,15,16,17,18] to parasitic infections [19]. However, their highly amphipathic nature and their dependence on lipid interactions is what also limits both structure/function investigations and the ability to target them in the case of intelligent drug design [20]. There is little information on the dynamic role of the membrane environment for any membrane protein, let alone PMPs. Predictably, this results in intense debate surrounding the mechanism of action or structure of many PMPs, leading to controversies in the field as in the case of type-II NADH dehydrogenases [21]. In this particular example, one active site is within the hydrophobic membrane interior while the other is in solution [21]. Most studies to date have only studied proteins such as this in the absence of lipids and their natural hydrophobic substrates—opting for solution-phase analogues instead. This leads to a disjoint between what is physiologically relevant, in terms of protein activity, and what can simply be recorded in vitro. For example, a recent study demonstrated that the F_1_F_o_ ATP synthase inhibitor bedaquiline (BDQ), while functioning as an inhibitor in solution, also acted as an ionophore in membranes [22]. In addition, a recent study demonstrated that an inhibitor that worked in solution against the type-II NADH dehydrogenase did not work when the same protein was embedded in a membrane [23]. 

There is clearly still a lot to learn about these proteins, with the influence of the lipid environment being consistently overlooked. Thus, it is not surprising that over 90% of promising drugs found using solution-phase studies are found to be ineffective in a physiological context. Here, we reflect on the diversity of MMPs/MAPs and PMPs, giving examples of key drug targets that are either from bacterial pathogens, or are directly involved in human disease (Table 1). In all cases, the membrane plays a role in function, yet this function is often poorly described. 

## 2. Monotopic Membrane Proteins (MMPs) and Membrane-Associated Proteins (MAPs)

### 2.1. Periplasmic Nitrate Reductase complex

The periplasmic nitrate reductase complex (Nap) is a well-described example of a MAP that associates with an MMP that is anchored inside the membrane [14], we refer to this as ‘the Nap complex’. The Nap complex has been characterized in multiple phototrophic and denitrifying bacteria, and is widespread among gram-negative bacteria. The primary function of this enzyme differs between species, and several physiological functions have been proposed. For example, while the Nap complex catalyzes the first step in aerobic denitrification, it has also been implicated in anaerobic respiration [45]. Although it is widely recognized that nitrate plays an important role in the environment, recent studies have also shed some light on its potential medical applications [15]. For many years, nitrite has been linked to carcinogenesis because it can form reactive N-nitroso compounds that are associated with cancer [14,15]. The formation of these toxic compounds has been argued to occur at non-physiological concentrations; for example, as a result of dietary intake. More recently, and despite the negative effects above, beneficial effects of nitrate and nitrite have been reported in the treatment of cardiovascular disorders and pulmonary hypertension [15]. Nitrate can be converted to nitric oxide, which serves as a pluripotent signaling molecule [15]. This signaling molecule is a key component in vasodilation and has numerous other physiological roles [15]. Given these functions, the concentration of nitrate and nitrite should be closely monitored to control various human diseases [15]. Of particular interest is their yet undescribed role in *Mycobacterium tuberculosis* infection [46], among other pathogens such as *Pseudomons aeruginosa* and *Haemophilus influenzae* (see Table 1).

**Figure 2:**
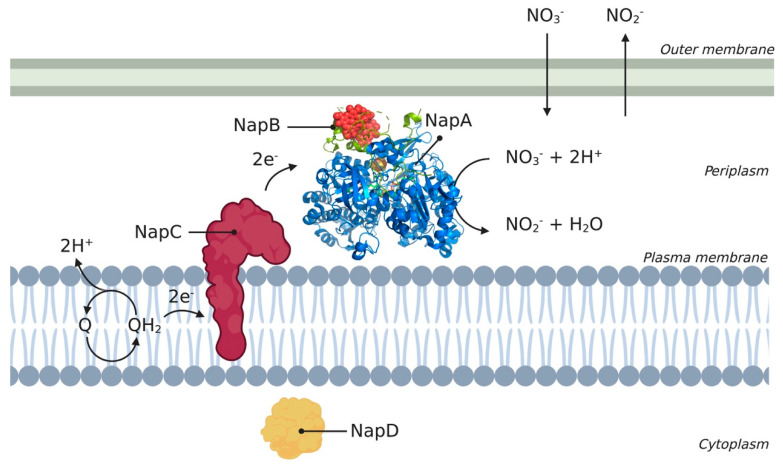
Schematic overview of Nap enzyme encoded from the nap operon. The different subunits (A, B, C, and D) are represented according to their location in the cell. Subunits NapA, NapB, NapC, and NapD are conserved across different species such as *Cupriavidus necator*, *Rhodobacter sphaeroides*, and *Thiospaera pantotropha*. The 3D structural cartoon of the *C. necator* NapAB (PDB:3ML1) [48] was rendered using PyMol and the figure compiled in BioRender.com [8].

The Nap complex is a heterodimer and consists of multiple subunits, of which NapA, NapB, NapC, and NapD are conserved among different species. A schematic overview of the different Nap complex subunits and their orientation in the cell is shown in Figure 2, where the periplasmic subunits catalyze the conversion of nitrate to nitrite using electrons supplied via the membrane-bound subunit. The Nap complex consists of a 90-kDa catalytic subunit, NapA, which contains a molybdenum cofactor and an N-terminal [4Fe-4S] cluster [47]. NapB is a 15-kDa bihaem cytochrome *c* and NapC is a 25-kDa membrane-bound tetrahaem cytochrome *c* (an MMP). NapA and NapB form a complex in the periplasm, with NapA providing the catalytic site and NapB functioning as an electron donor to NapA. NapC is anchored inside the membrane using a hydrophobic α-helix and is thought to be involved in electron transfer between the membrane quinol pool. The link between the quinol pool and the soluble reductases is the membrane anchor of NapC [14]. NapD is located in the cytoplasm and plays a role in the maturation of NapA [45]. The Nap complex does not directly contribute to the generation of a proton motive force (PMF) and is also independent of the energy-conserving cytochrome *bc*_1_ complex [14].

### 2.2. CymA

CymA is a monotopic membrane tetrahaem-containing cytochrome *c* (an MMP), belonging to the NapC/NirT superfamily. It is present in a wide variety of pathogenic gram-negative bacteria, but is best described from the inner membrane of the γ-proteobacterium *Shewanella oneidensis* (*S. oneidensis*), a facultative anaerobe [49,50].

In these pathways, CymA catalyzes the two electron reduction of membrane-bound quinones to QH_2_ [51,52]. However, in contrast to NapC/Nir, CymA is much more promiscuous in its interactions with MAPs and is the key point of membrane interaction for several soluble terminal reductases [52,53]. This central hub-like role makes CymA an interesting enzyme to investigate in various pathogens, since inhibiting the enzyme would result in a significant loss of electron transport chain function, compromising the ability of the pathogen to live and adapt [54]. 

**Figure 3:**
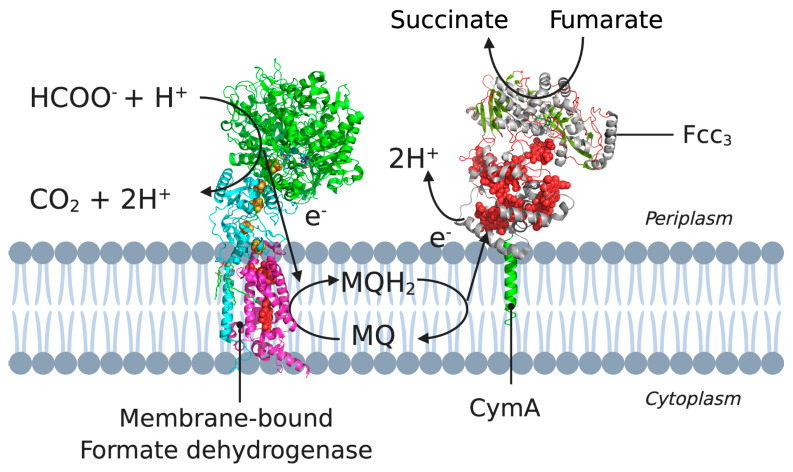
A cross-section of the cytoplasmic membrane of *S. oneidensis* showing the electron pathway for the reduction of CymA. Example pathway starting from formate oxidation (by formate dehydrogenase) to the electron transfer between MQH_2_ and CymA, to NapAB. 3D structural cartoons of the *Escherichia coli* formate dehydrogenase (PDB:1KQG) [56], the *Desulfovibrio vulgaris* NrfH (PDB:2J7A) [5], and the *Shewanella frigidimarina* flavocytochrome *c*_3_ (PDB:1Y0P) [6] were rendered with PyMol and the figure compiled in BioRender.com [8].

CymA has a single N-terminal transmembrane α-helix and a periplasmic globular domain containing four *c*-type haems, three low-spin and one high-spin [52]. In these pathways, CymA transfers electrons from menaquinone-7 (MQ-7) to several distinct terminal reductases [52,55]. An overview of the electron pathway is shown in Figure 3.

Early investigations suggested that the reduction of CymA could occur either directly, using QH_2_, or indirectly, using an integral membrane protein [53]. However, it has since been revealed that not only was the QH_2_ binding site unambiguously located in the globular domain of CymA, but also that CymA directly interacts with MQ-7 in the lipid phase [52,55]. The purification of CymA and MQ-7 as a complex and the catalytic behavior of CymA indicate that MQ-7 is tightly bound in a binding pocket of CymA, making MQ-7 a functional cofactor [55]. The membrane environment plays a significant role in the guidance of MQ-7 from the membrane to the binding pocket in CymA. McMillan et al. (2012) proposed that the tight binding of MQ-7 to CymA facilitates the pass-off of electrons from the haem groups in CymA to different quinones in solution or in the membrane. In this reaction scheme, MQ-7 first binds with high specificity in a binding pocket of CymA and gives off its electrons to the haem groups in CymA. The binding of MQ-7 then makes the subsequent binding of other quinones possible in a less specific and weaker active site pocket. These quinones receive the two electrons initially coming from MQ-7 [55]. 

Interestingly, later research showed that CymA does not oxidize MQ-7 in the absence of a partner protein [57]. Since CymA needs to form a complex to trigger MQ-7 oxidation, the respiratory electron flux is therefore dependent on the lifetime of the complexes formed [58]. The electrons retrieved from the oxidation of MQ-7 are distributed to the partner protein by CymA, to eventually end up in fumarate, nitrate, nitrite, iron, and extracellular DMSO [57].

### 2.3. Membrane-Anchored Cytochrome c and a Paradigm Change

In the classical models of photosynthetic and respiratory electron transfer, a soluble, freely diffusible protein functions as an electron carrier between the oxygen or photochemical reaction centers and the *bc*/*bf* oxidoreductases. This soluble protein was often described as a *c*-type cytochrome (cyt), also simply ‘cyt *c*’. According to these classical models, the membrane proteins that participate in the electron transfer are not directly ‘hard-wired’ to each other, but rely on the connections laid by water-soluble and lipid-soluble electron carriers such as cyt *c* [59]. This model became the standard until, in the 1980s, a gene was identified that transcribed a membrane-associated cyt *c*, which was named cyt *c_y_.* This protein has an unusual bi-partite structure and is characterized by a membrane-anchor, extended by a large, flexible stretch of amino acids in a structure not dissimilar to CymA [60]. This stretch of amino acids functions as an amino terminal ‘anchor-linker’ domain, connecting the membrane anchor to the cyt *c* domain [59]. 

In gram-negative, purple, non-sulfur, facultative phototrophic bacteria such as *Rhodobacter capsulatus* (*R. capsulatus*), the cytochrome *bc*_1_ complex donates electrons to both soluble cyt *c*_2_ and membrane-anchored cyt *c*_y_. In studies performed by Myllykallio et al. (1998) on the speed of the electron transfer by the two cyt *c* species, it became clear that the membrane-anchored cyt *c*_y_ electron transfer pathway was more efficient, especially during multiple turnovers [61]. This is probably due to the close proximity of the photochemical reaction center, cyt *c*_y_, and the cyt *bc*_1_ complex, which enables the significantly faster re-reduction of cyt *c*_y_ by the *bc*_1_ complex. The close proximity of these complexes also suggests the formation of electron-transfer super-complexes that enhance electron transfer via substrate channeling [62]. 

## 3. Glycosylphosphatidylinositol (GPI)-Anchored PMPs

A special subset of anchored membrane proteins are glycosylphosphatidylinositol (GPI)-anchored proteins. These proteins are not anchored inside the membrane with a hydrophobic segment, but are covalently attached to the head group of lipids. A GPI-anchored protein is attached to the lipid phosphatidylinositol (PI) at the carboxyl terminus by a post-translational modification. This GPI anchor represents a post-translational modification of proteins with a glycolipid [63]. They are generally found in the outer leaflet of the lipid bilayer, facing the extracellular space [64]. The structure of the protein–carbohydrate association in GPI-anchored proteins is unique in the way that the reducing terminus of the GPI oligosaccharide is not attached to the protein, but to the lipid. First, the GPI anchor is assembled on a phosphatidylinositol (PI) lipid on the endoplasmic reticulum (ER) membrane by a series of enzymatic reactions [63]. The reducing terminal glucosamine residue is α1-6 linked to the D-*myo*-inositol head group of a phosphatidylinositol (PI) moiety. At the other end, a distal mannose residue is attached to the protein. In this linkage an ethanolamine phosphate (EtNP) bridge is formed between the C-6 hydroxyl group of mannose and the α-carboxyl group of the carboxy-terminal amino acid [64]. These two linkages result in the indirect binding of a protein to the PI lipid using a GPI anchor, resulting in a mature GPI-anchored protein. To determine what proteins get attached to a GPI anchor, the carboxyl terminus of all GPI-anchored proteins contains a hydrophobic signal sequence that triggers the addition of a GPI anchor. While adding the GPI anchor, this signal sequence is cleaved off and replaced with the preassembled anchor via a transamidation reaction. The GPI anchor with attached protein and lipid is then further modified in the ER and Golgi apparatus, and subsequently transported to the cell surface [63]. 

**Figure 4:**
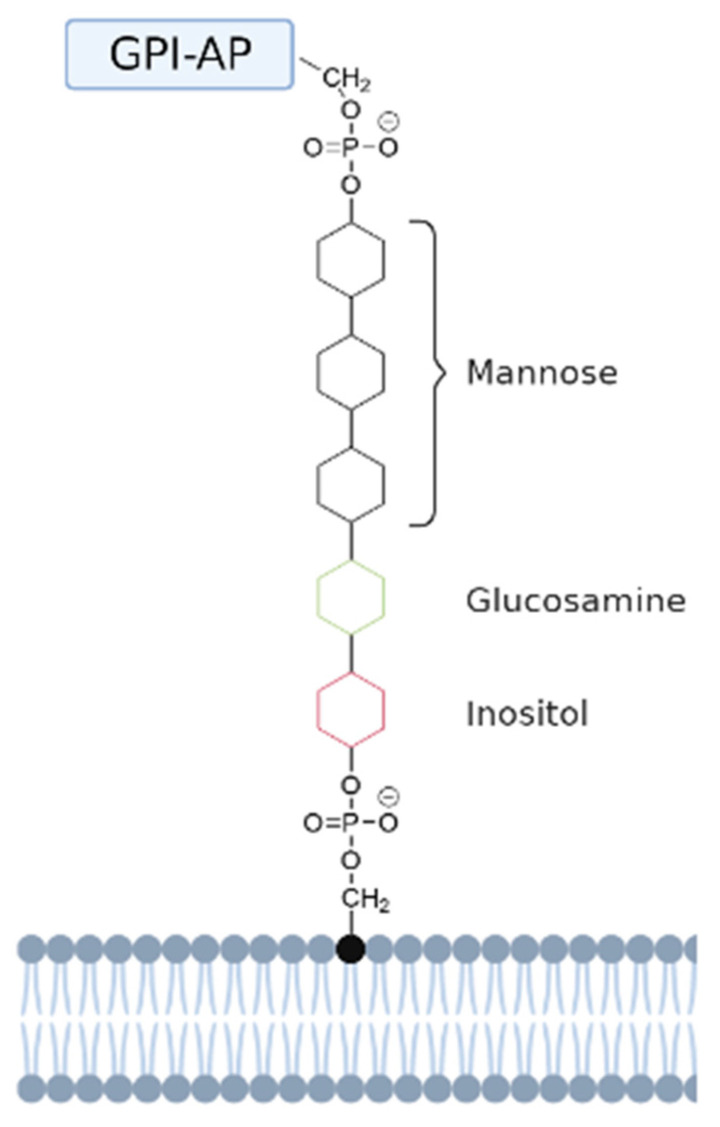
Schematic representation of a GPI-anchored protein. The GPI-anchored protein (GPI-AP) is anchored to the head group of a phosphatidylinositol (PI) lipid via a GPI anchor. This anchor has a conserved core domain consisting of ethanolamine-PO_4_-6Manα1-2Manα1-6Manα1-4GlcNα1-6*myo*-inositol-1-PO_4_-lipid [64]. The mannose, glucosamine, and inositol groups are represented by black, green, and red aromatic rings, respectively.

GPI-anchored lipids have a core structure that consists of the following sequence: ethanolamine-PO_4_-6Manα1-2Manα1-6Manα1-4GlcNα1-6*myo*-inositol-1-PO_4_-lipid (see Figure 4).

Aside from the core structure, GPI-anchors are very diverse, depending on the protein they are anchored to and the organism that synthesizes them [64]. Once in the correct position, GPI-anchored proteins have a variety of roles, including transporters, adhesion molecules, receptors, enzymes, protease inhibitors, and migration molecules [63,65]. 

### 3.1. Alkaline Phosphatase 

Alkaline phosphatases (AP) are typical GPI-anchored proteins, belonging to a family of ectonucleotidase enzymes that play an important role in regulating the availability of nucleotides, which serve as extracellular signaling molecules. Controlling the availability of these signaling molecules would open up a great ability to modulate certain cellular processes. In humans, APs are present in all tissues but are especially concentrated in the liver, kidney, and bones. Thus, APs are seen as very interesting drug targets [34]. Through their phosphodiesterase, phosphohydrolase, and transphosphorylase activities, APs can hydrolyze a wide spectrum of substrates, including pyrophosphates and phosphatidates. As ectonucleotidases, they are responsible for hydrolyzing extracellular nucleotides to nucleosides, and are for this reason able to convert ATP, ADP, and AMP to adenosine [66]. These nucleosides act on their respective receptors, such as the adenosine-activated P1 receptor, and by doing so trigger cellular responses that lead to physiological and immunological changes. The production of adenosine is the hallmark that proves ectonucleotidases are involved in regulating the purinergic cell signaling pathway [34]. In the purinergic cell signaling pathway, purine nucleotides and nucleosides, such as adenosine, serve as extracellular signaling molecules that affect their dedicated receptors and trigger a cellular response. Adenosine 5′-triphosphate (ATP) has been shown to be a co-transmitter in all nerve types in both the peripheral and central nervous systems. In addition, purines are extracellular messengers to non-neural cells, including secretory, inflammatory, and endothelial cells. Separate membrane receptors for adenosine and ATP were identified in 1978, and are called, respectively, P1 and P2 receptors. P2 receptors can be divided into P2X and P2Y receptors, of which seven and eight subtypes are currently known, respectively [67,68]. 

APs are homodimeric metalloenzymes with two Zn^2+^ ions and one Mg^2+^ ion in their active site to aid in catalytic function, and one Ca^2+^ ion at a non-catalytic site [66]. APs can be divided into two categories: tissue-nonspecific alkaline phosphatases (e.g., those found in the intestine, placenta, and germinal tissue) and tissue-specific alkaline phosphatases (TNAP) [69]. APs are located on the surface of plasma membranes or secretory vesicles and are, as previously mentioned, anchored to the cell surface by a GPI anchor. Once on the membrane, APs are localized to lipid rafts and are consequently part of dynamic assemblies of sphingolipids and cholesterol that associate/dissociate on a sub-second timescale [66]. These rafts are in a liquid-ordered phase and have characteristically ordered phospholipid chains. Within these rafts, lipids and proteins generate the compartmentalization of cellular processes, and are involved in processes such as signal transduction and membrane trafficking [70]. 

In the formation of calcifying tissues such as bones and teeth, proper mineralization is fundamental to support precipitation of calcium phosphate. APs, and more specifically TNAPs, aid in the mineralization process by providing a localized phosphate pool, using its non-substrate-specific phosphatase activity. Overexpression of TNAPs result in disorders in bone formation, such as unwanted disposition of calcium phosphate in soft tissues, and gives rise to hydroxyapatite deposition disorder. To regulate mineralization and prevent diseases such as hydroxyapatite deposition disorder, the inhibition of TNAP has become a field of interest for drug targets [34]. 

### 3.2. Ecto-5′-Nucleotidase (CD73)

**Figure 5:**
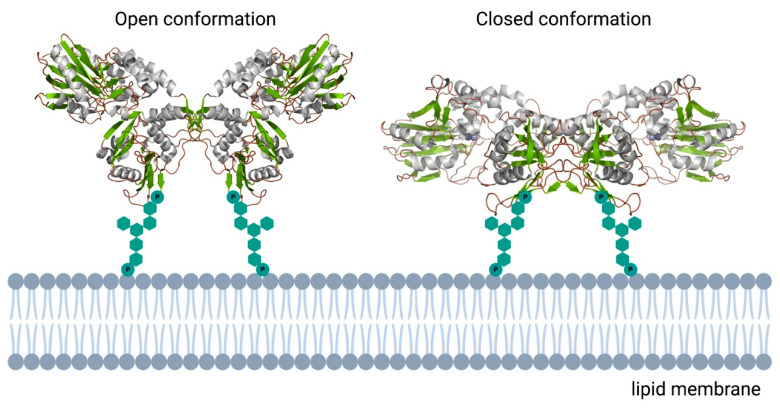
Schematic overview of the human ecto-5′-nucleotidase. In contrast to the monomeric bacterial enzymes, eukaryotic e5NT exists and functions as a non-covalent homodimer. Open and closed conformations represented as reported in [73]. 3D structural cartoons of the *Homo sapiens* CD73 (PDB:4H2B and PDB:4H2I) [74,75] were rendered with PyMol and the figure compiled using BioRender.com [8].

Ecto-5’-nucleotidase, also called CD73, is an alkaline phosphatase-like protein involved in the hydrolysis of extracellular nucleotides to adenosine. Ectonucleotidases can thus regulate the purinergic cell signaling pathway. In contrast to common APs, CD73 can only convert AMP to adenosine. ATP and ADP are both competitive inhibitors, binding to the active site without being hydrolyzed. Consequently, in the presence of extracellular ATP or ADP, CD73 depends on APs to regain activity [71]. CD73 plays a major role in several pathologies, ranging from cardiovascular disease to cancer. However, the required activity and treatment differs depending on the clinical picture. A schematic overview of the mechanism of CD73 is presented in Figure 5. The retention of nucleotides and decrease in the production of adenosine associated with CD73 inhibition may have severe effects and could be an important cause of various pathologies, such as atherosclerosis and thrombosis [72]. A reduced activity of CD73 increases the permeability of the endothelium, leading to enhanced leukocyte transmigration.

Upregulation of CD73 may, therefore, be protective to a number of cardiovascular diseases, and causes this enzyme to be of great interest in treatment strategies for endothelial protection [72]. In addition to inhibition, overexpression of CD73 has been shown to be associated with the altered ability of cells to adhere and/or migrate [76]. Since this phenomenon is present in cancer cells, inhibitors of CD73 are being considered for use in the therapies of melanomas, gliomas, and breast cancer [16]. 

A mature ecto-5′-nucleotidase consists of 548 amino acids and has a stretch of about 25 amino acids at the C-terminus that is replaced by a GPI anchor. Using this GPI anchor, the mature protein is attached to the cell membrane with the C-terminal serine residue linked to oligoglycan and sphingolipidinositol groups. The C-terminal domain of CD73 provides the binding pocket for AMP, and the N-terminal domain oversees the binding of two catalytic metal ions, Zn^2+^ and Co^2+^ [71,72]. 

As a result of its high abundance in endothelium, CD73 has a broad distribution across the human body and can be found in the kidney, liver, heart, lungs, and brain [72]. Inside the endothelium, the major function of CD73 is the regulation of adenosine concentration by hydrolyzing AMP [72]. As mentioned previously, adenosine is the ligand for specific receptors located in the membrane called G-protein coupled receptors (P1). There are four known subtypes of the P1 receptors in humans called A_1_, A_2A_, A_2B_, and A_3_, numbered according to their affinity for adenosine [34]. To function in cell physiology, CD73 and the A_1_ receptor are located in lipid rafts, where they can interact. The lipid raft gives CD73 a high mobility, and facilitates its ability to shuttle to A_1_ and form a complex [77].

Through its receptor, adenosine regulates the migration of lymphocytes through the endothelial cell barrier. In healthy tissue, it prevents the infiltration of leukocytes and in this way protects the tissue from inflammatory damage. Moreover, extracellular adenosine prevents the differentiation of monocytes into macrophages and inhibits their phagocytic action. With lower CD73 activity, less adenosine is available to trigger P1 receptors. This makes the endothelium more permeable and causes endothelial injury as a result of the unwanted inflammatory reaction. Endothelial injury disrupts the vascular homeostasis and causes vessel wall narrowing, thrombosis, and oxidative stress, and is therefore seen as one of the most important mechanisms in the pathogenesis of cardiovascular diseases [72].

### 3.3. Acetylcholinesterase

Acetylcholinesterases (AChE) are serine hydrolases that are primarily found at postsynaptic neuromuscular junctions in muscles and nerves [78]. AChE hydrolyses acetylcholine (ACh) into acetic acid and choline. ACh is a naturally occurring neurotransmitter that is released when a neural signal propagates and binds to an ACh receptor on a cellular membrane. The downstream effect of a signal initiated by ACh is the amplification and propagation of a cellular signal. By breaking down ACh, the primary goal of AChE is to terminate neuronal transmission and signaling between synapses to prevent ACh dispersal and unwanted activation of nearby receptors [78]. 

AChEs are inhibited by organophosphates, which are one of the most common causes of poisoning in the world as a result of organophosphate ingestion by agricultural, accidental, or deliberate exposure [78]. Moreover, irreversible AChE inhibitors are also commonly used in biological warfare [78]. However, controlled treatment with AChE inhibitors can provide some therapeutic relief to Alzheimer’s disease patients by creating a localized overexpression of AChE. This excess AChE leads to increased stimulation of nicotinic receptors and increases the possibility of a correctly propagated signal [78,79]. 

AChE is derived from a single mammalian gene, but in physiology, tends to occur in multiple forms. These forms are the result of alternative mRNA splicing and distinct modes of subunit assembly or post-translational modification. All splice variants possess the same catalytic domain, but differ in small C-terminal domains. The AChE_R_ variant produces a soluble monomer which is upregulated in the brain during stress. The erythrocyte AChE (AChE_H_) is a dimer and is anchored to the membrane by a GPI anchor [80]. AChE_T_ is the only AChE that is expressed in the brain and muscles of mammals. This variant can exist in various forms including monomers, dimers, and tetramers. They can also have collagen or hydrophobic tails, with which they are anchored in the membrane [79]. AChE_H_ and AChE_T_ polymerize into dimers using a disulfide bond between two cysteine residues [78]. AChE_H_ contains, like many other GPI-anchored proteins, an additional fatty acid (e.g., palmitic acid) on the 2-hydroxyl of the inositol ring. This extra fatty acid renders the protein resistant to cleavage by phosphatidylinositol phospholipase C, which can otherwise cleave the GPI-anchor [81]. 

## 4. Membrane-Binding PMPs

In place of a membrane anchor, PMPs also interact with the membrane via electrostatic interactions with the polar head groups of phospholipids, or by horizontal helices that allow hydrophobic interactions if the PMP is ‘nestled’ into the lipid leaflet. 

### 4.1. Alternative Oxidase

Alternative oxidases (AOXs) are membrane-associated terminal quinol oxidases found in the respiratory electron chains of a variety of species across different kingdoms [82]. Since AOXs have no human homologue, they been investigated intensively as the drug target for pathogenic fungi and the human parasite *Trypanosoma brucei* (*T. brucei*) [19]. African trypanosomiasis, or sleeping sickness, is caused by *T. brucei* parasites, and is fatal if left untreated. In these organisms, AOXs play a critical role in their energy metabolism, and have thus become a novel therapeutic target in the treatment of diseases caused by these pathogens [19]. 

AOXs catalyze the cyanide-resistant reduction of oxygen to water without the translocation of protons across the inner mitochondrial membrane [83]. In doing so, they function as a non-energy-conserving component of the respiratory electron transport chain [83]. In early work on AOXs, it was revealed that AOXs need iron to function properly [82,84]. Therefore, the requirements for AOXs to be active can be summarized as follows: they must interact with oxygen, with a membrane, with quinols, and with iron [82]. Crystal structures of AOXs showed that these enzymes are homodimers, with each monomer being comprised of six α-helices. Four of these α-helices form a 4-helix bundle, which acts as a scaffold to bind two iron atoms. This 4-helix bundle with a diiron center forms the active site of the enzyme. In earlier work performed by Siedow et al., (1995), it was suggested that AOXs are anchored inside the membrane using two transmembrane helices [85]. However, Andersson and Nordlund refined this model after the discovery of highly conserved glutamine and histidine ligands that aid the binding of the two iron atoms in the active site. In this refined model the two transmembrane helices also formed part of the active site and were not transmembrane helices, but were involved with membrane association [86]. Consequently, the alternative oxidases were proposed to interact with one leaflet of the membrane bilayer [83]. Using ribonucleotide reductase as a basis for the Andersson and Nordlund model, they observed that two conserved residues, Q247 and Y258, exist in a crevice leading from a hydrophobic (possibly membrane-binding) region towards the diiron center between α helices 2 and 3 of the AOXs [82,83]. Thus, this hydrophobic region might form a monotopic interaction with the membrane. 

In similar membrane proteins (e.g., prostaglandin synthase isozyme-1), this hydrophobic plateau is encircled by positively charged residues positioned in an ideal way to interact with the head groups of phospholipids. These positively charged residues might be the conserved residues, H198 and R203, that are positioned between helix 1 and 2 in the AOXs. They have been proposed to play a role in membrane association using their positive charges for binding of the phosphate groups on the membrane [82,83]. Finally, the alternative oxidases may supply membrane interactions using the conserved residues H266, G270, E274, A275, and Y280 in α helix 3 [82]. 

### 4.2. Cytochrome c

**Figure 6:**
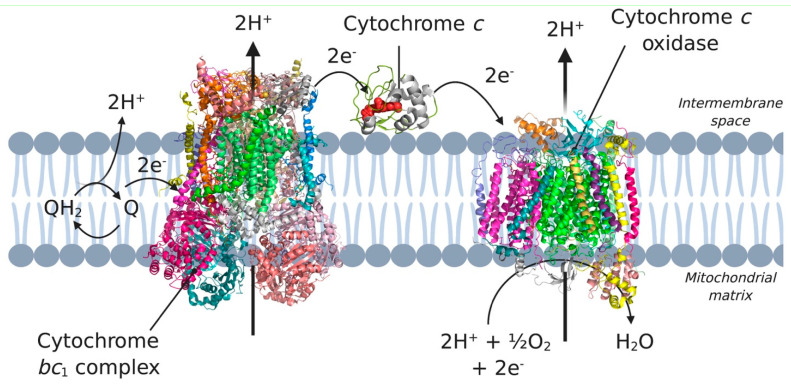
Complex III, complex IV and cytochrome *c* in the electron transport chain. Electrons coming from complex I and II are transferred to ubiquinone (Q) in the membrane. Subsequently, the electrons are transferred to complex III, cytochrome *c*, and complex IV, which eventually incorporates the electrons in water. 3D structural cartoons of the *Homo sapiens* cytochrome *bc*_1_ (PDB:5XTE) [89], the *Homo sapiens* cytochrome *c* oxidase (PDB:5Z62) [90], and the yeast cytochrome *c* (PDB:4Q5P) [91] were rendered using PyMol and the figure compiled using BioRender.com [8].

As mentioned previously, in addition to the membrane-anchored cyt *c*, a soluble variant of cytochrome *c* (cyt *c*) is found in nature. Cyt *c* is a small, highly basic hemoprotein found in the intermembrane space of mitochondria (cytoplasmic membrane of bacteria) which has two biological functions in human mitochondria [87]. The core biological function of cyt *c* is to catalyze the transfer of electrons from cyt *c* reductase, the *bc*_1_ complex, to the IMP cytochrome *c* oxidase (COX; see Figure 6). This reaction step begins with diffusion of the soluble cyt *c* towards the membrane bound COX, which is facilitated by long-range electrostatic attraction between the two proteins, cytochrome *c*, and the membrane [88]. 

The second biological function of cyt *c* is the activation of the apoptotic pathway through cyt *c* release from mitochondria into the cytosol. When cyt *c* binds to the mitochondrial inner membrane, it not only facilitates electron transfer in the respiratory chain, but also prevents the cell from going into apoptosis [87]. This release of mitochondrial cyt c has long been proposed to be via the permeability transition pore.

Interestingly, the dimeric F_1_F_o_ ATP synthase, part of the respiratory chain that cyt *c* is part of, has been implicated in the permeability transition pore, a recent finding that may help shed light on the release of cyt *c* from mitochondria [92].

However, cyt *c* is also an interesting target in, for example, cancer treatment because it activates the apoptotic pathway through cyt *c* release from mitochondria into the cytosol. Targeted cyt *c* delivery to cancer cells is now considered a potential treatment to induce apoptosis and thereby kill the cancer cells [17].

As was previously mentioned, cyt *c* needs to be attracted to the membrane in order to fulfil its core biological function. This attraction is facilitated by the lipids constituting the mitochondrial membrane, and in particular by cardiolipin (CL) [93]. CL is a structurally unique phospholipid containing a small head group but four acyl chains. This phospholipid is a major component of the mitochondrial inner membrane, and it is indispensable for protein retention, stability, and normal functioning [87]. Deprotonated or partially protonated cardiolipin forms a complex with cyt *c* via electrostatic interactions, but possibly also through hydrogen bonding and hydrophobic interactions [93]. The interaction of this unique lipid with cyt *c* is predominantly located at two acidic phospholipid binding sites on the surface, called the A- and C-sites. The A-site is suggested to be involved in electrostatic interactions of deprotonated CL molecules with cyt *c*, and the C-site is responsible for protein binding to protonated phospholipids using hydrogen bonding. The interactions between CL and cyt *c* not only determine the location of cyt *c* association with the membrane, but also cause conformational changes of the protein upon its association with lipids and protein-induced structural rearrangement of the lipid bilayer [93].

Together with the unique structural features of CL, a hydrophobic crevice was discovered in the tertiary structure of cyt *c* [93]. This led to the suggestion that upon binding to the membrane, a so-called ‘extended lipid conformation’ could be formed. In this conformation, lipid acyl chains point in the opposite direction of the head group, generating a straight angle of 180°. One acyl chain of CL remains in the lipid bilayer and another extends outward. This extending acyl chain is then embedded in the hydrophobic crevice of cyt *c*. This anchorage is hypothesized to cause conformational changes in cyt *c*, such as loosening of the tertiary structure, alterations in the haem environment, and reversible unfolding [87,93]. Binding of cyt *c* to the membrane causes a modulation of the lipid composition in the inner mitochondrial membrane. Specifically, cyt *c* binding induces changes in CL distribution, leading to the formation of CL-rich microdomains. Multiple studies have shown that cyt *c* entrapment in a CL domain enhances the rate of electron transfer by the protein, and that CL domains may lead to amplification of the apoptotic signal [87]. 

### 4.3. Type-II NADH Dehydrogenase 

Type-II NADH dehydrogenases (NDH-2s) are NADH:quinone oxidoreductases that play a major role in the respiratory metabolism of bacteria and in the mitochondria of fungi, plants, and protists [94]. They catalyze the two-step electron transfer from an aqueous NADH substrate, via their cofactor FAD, to a membrane-bound quinone substrate, most commonly ubiquinone and menaquinone. In contrast to the type of NADH dehydrogenases present in humans (i.e., type-I NADH dehydrogenases or respiratory complex I), type-II NADH dehydrogenases do not pump protons across the membrane, contributing only to membrane electrical potential (Δψ) [95]. They have no human homologue, so make for excellent drug targets against a wide variety of pathogens from *Staphylococcus aureus* to *Mycobacterium tuberculosis* (see Table 1). 

These enzymes have been described as peripheral membrane proteins, located in the inner mitochondrial membrane facing the cytosol, or in the cellular membranes of bacteria. Their mode of interaction with the membrane remains a point of discussion. Multiple researchers have suggested interactions, ranging from electrostatics to two anchoring in the membrane via hydrophobic interactions with amphipathic α-helices that span the entire protein [96,97]. 

To further analyze this interaction, Villegas et al. (2011) produced a truncated version of NDH-2 without the last 43 amino acids. This C-terminal region is suggested to play a role in the interaction of NDH-2s with the membrane, and the truncated version was therefore believed to have lost the interaction with the membrane [98]. From the work of Villegas et al. (2011), it became clear that most of the truncated protein fraction was located in the cytosol, compared with the wild type where it was located in the membrane fraction [95]. These results, together with the finding that mild non-detergent treatment releases NDH-2s from the membrane fraction, indicates that NDH-2s do not have transmembrane helices that anchor the protein in the membrane, but that a C-terminal region is involved in this interaction. The C-terminal fragment Arg390-Ala405 is sufficient to bind the protein to the lipid membrane and also contains several amino acids that are highly conserved amongst NDH-2 species. The hydrophobic and hydrophilic amino acids are located on opposite sides of the predicted α-helix, suggesting a membrane–protein interaction through the hydrophobic face of this amphipathic α-helix [95]. 

Kinetic characterization of NDH-2s has provided insight into their catalytic mechanism, yet the exact mechanism remains hotly debated. On the one hand, some evidence suggests that they follow a ping-pong mechanism, in which the two substrates bind, react, and dissociate sequentially [99]. In such a scenario, the substrates can either have the same binding site (one-site ping-pong mechanism) or distinct binding sites (two-site ping-pong mechanism) [13]; always considering that, in a ping-pong mechanism, the substrates are never bound simultaneously. Alternatively, some researchers have observed the formation of charge-transfer complexes, which are formed by intermediate states between NADH/oxidized flavin and NAD^+^/reduced flavin. This finding suggests that the charge-transfer complex is oxidized by quinone, and that the two substrates are bound to the enzyme at the same time [99]. Since the formation of a stable charge-transfer complex suggests that NADH oxidation has already occurred to some extent, this scenario is indicated as an atypical ternary complex mechanism [99]. Recently, Godoy-Hernandez et al. (2019) revealed the membrane-bound catalytic oxidation of NADH by *Caldalkalibacillus thermarum* NDH-2 (*Cth*NDH-2) using a novel bioelectrochemical platform to address the membrane-bound quinone [21]. This study suggested that two MQ-7 molecules native to *Caldalkalibacillus thermarum* bioenergetics [100,101] might interact, with each involving two different quinone-binding sites: one Q_I_ site, closer to the FAD, and another more distal Q_II_ site that is presumably shaped by the membrane lipids, as well as the isoprenoid tail of the quinone substrate. Interestingly, this structural hypothesis was further supported by earlier crystallographic evidence. On the one hand, the crystal structure of the *S. cerevisiae* with UQ_4_ showed two occupied quinone sites (i.e., Q_I_ and Q_II_, respectively [102]), whereas the same crystal structure with UQ_2_ showed a single occupied site (i.e., Q_I_ [103]). Finally, the authors proposed a co-operative mechanism that involved two quinone-binding sites, which are both lipid- and isoprenoid-dependent [21].

### 4.4. Dihydroorotate Dehydrogenase 

**Figure 7:**
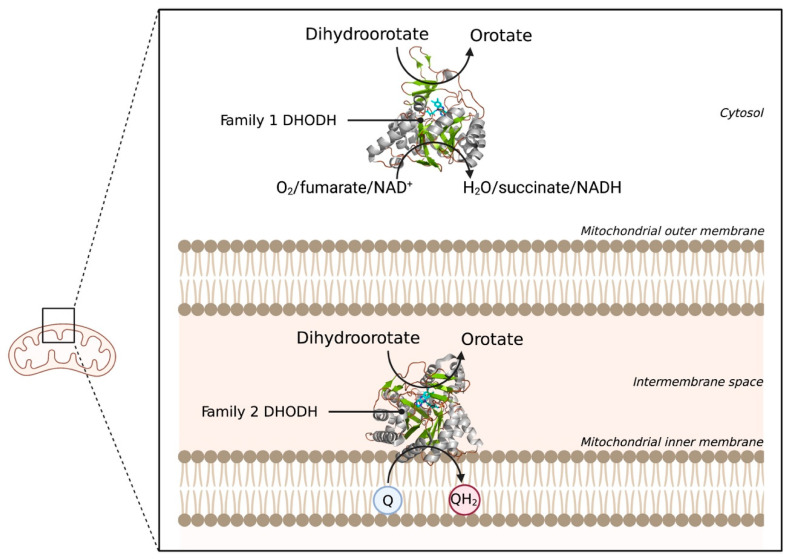
Schematic overview of the locations and reactions of both DHODH families. The two DHODH families are visualized with their respective location in the cell. Family 1 DHODH uses O_2_, fumarate, or NAD^+^ as electron acceptors. Family 2 uses ubiquinone (Q) as an electron acceptor, which gets reduced to QH_2_. 3D structural cartoons showing the Family 1 DHODH from *Trypanosoma brucei* (PDB:5XFV) [106], and the Family 2 DHODH from *Plasmodium falciparum* (PDB:6VTN) [107] were rendered with PyMol and the figure compiled using BioRender.com [8].

The last example of an electrostatically associated peripheral membrane protein is the enzyme dihydroorotate dehydrogenase (DHODH), which has been revealed as a potential target in the fight against cancer. DHODH is involved in de novo pyrimidine biosynthesis, necessary to construct DNA and RNA and thus multiply cells. In healthy cells, the de novo pyrimidine biosynthesis route is not much used, but in rapidly proliferating cells, such as tumor cells, this pathway is the number one supply of pyrimidines. Inhibition of DHODH could therefore potentially lead to specific depletion of pyrimidines in tumor cells. However, whether the depletion of pyrimidines could trigger the response of the p53 tumor suppressor, and ultimately lead to p53-mediated cell death, is still an open question [18]. DHODHs can be classified into two families, family 1 and 2. Family 1 DHODHs are cytosolic and use oxygen, fumarate, or NAD^+^ as electron acceptors. Family 2 DHODHs are membrane-bound and use ubiquinones as electron acceptors. A schematic overview of the location and reactions of both families can be seen in Figure 7. DHODH catalyzes two redox reactions: the oxidation of dihydroorotate to orotate and the subsequent ubiquinone reduction [104]. The oxidation of dihydroorotate is a stepwise mechanism with highly conserved residues. DHO is deprotonated at the C5 position using a catalytic base residue, after which a hydride is transferred to a flavin mononucleotide (FMN). In the second reaction, a ubiquinone from the electron transport chain is needed to regenerate FMN, via a two-electron transfer [105]. The electrons pass via the co-factor FMN to the membrane-bound acceptor ubuquinone-10 (Q10). Therefore, targeted occupation of the coenzyme Q10 binding site in the amphipathic domain can be used to inhibit DHODH [20].

Membrane association occurs via the N-terminal region, which contains a mitochondrial signal sequence, a putative transmembrane helix, and two amphipathic helices. The amphipathic region is the membrane-binding domain that facilitates the two-electron transfer of the soluble substrate dihydroorotate. This amphipathic region is involved in lipid interactions to position the protein and bind it to the membrane.

To determine which lipids contribute to this interaction, Costeira-Paulo and co-workers (2018) performed an assay using the three most common lipids in the mitochondrial membrane: phosphatidyl ethanolamine, phosphatidyl choline, and CL [20]. From this experiment, the researchers concluded that the protein had formed a protein-lipid complex at low concentrations of CL and phosphatidyl ethanolamine, indicating a high affinity for these lipids. Since the lipids have similar acyl groups, the difference in interaction is caused by a preference for anionic head groups that can provide charge interactions. The lipids are bound via salt bridges formed between the negatively charged phosphate moieties of the head groups and positively charged side chains of DHODH. Upon further investigation, it was discovered that not only do the N-terminal amphipathic helices (residues 35–51 and 52–67) bind to the charged lipid head groups, but the soluble domain also interacts with the membrane. By extending the side chains of the surface residues K167 and R162 towards the lipid head groups, the amphipathic helices of DHODH are partially lifted off the membrane [20].

Although the catalysis of the human DHODH has been studied in solution and in the presence of detergents, its mechanism in the enzyme’s native membrane environment has yet to be revealed. Provided that DHODH is a critical target for drugs against cancer, knowledge of this mechanism could ultimately prove fundamental to applications in DHODH-targeted therapy.

## 5. Conclusions and Outlook

Peripheral membrane proteins (PMPs) are a highly diverse group of proteins that can interact with the phospholipid bilayer in unique ways: through direct binding using hydrophobic alpha helices or GPI anchors, or indirect binding using electrostatic and hydrophobic interactions. The method of interaction with the membrane influences the function of the proteins, and can dictate if a protein can form a shuttle between the cytoplasm and the membrane, as is the case in cyt *c*. Not only their unique amphipathic nature, but also their seemingly key role in numerous diseases altogether makes these enzymes highly relevant research targets. As discussed in the introduction, the mechanism of action or structure of many PMPs is currently under debate, which can sometimes even lead to controversies in the field, indicating that there is still a lot to learn about these proteins, and that the influence of the lipid environment is often overlooked. 

**Figure 8:**
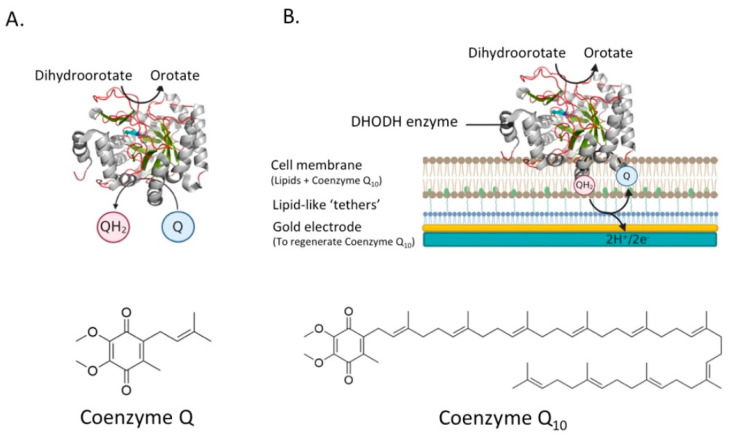
The reactions catalyzed by DHODH and a schematic overview of the proposed system to analyze the activity of DHODH. (**A**) DHODH catalyzes the oxidation of dihydroorotate (DHO) to orotate. The two electrons that are removed in that reaction are transferred to coenzyme Q via a flavin mononucleotide (FMN). (**B**) The activity of DHODH could be monitored using a planar tethered lipid bilayer system, homologous to that reported in Godoy-Hernandez et al., (2019). In this system, a gold electrode is connected to a cell membrane containing the DHODH enzyme. QH_2_ that is generated after the reduction of coenzyme Q by DHODH is regenerated at the gold electrode. The two electrons coming from that reaction will generate a current in the electrode. 3D structural cartoons showing the *Homo sapiens* DHODH (PDB:2PRH) [109] were rendered using PyMol and the figure compiled using BioRender.com [8].

For example, taking the catalysis of human DHODH as an example, catalysis in the enzyme’s native membrane environment has yet to be revealed. Novel planar bilayer techniques are being developed that take into account these considerations, which are more extensively reviewed by Rossi and Chopineau and indeed represent an ever-expanding area [108]. For example, we use a platform extremely well-suited to research involving quinone-utilizing proteins [21,51], but can easily be extended to other proteins, especially when used in combination with a quartz-crystal microbalance (QCM-D) [57]. In our system, a planar tethered lipid bilayer system, in which a gold electrode is connected to a cell membrane containing the enzyme, is extremely accurate in measuring the electrons involved in the reaction (see Figure 8B). In this system, the proteins can be investigated in their native environment, which is key when testing the inhibitory effect of potential drug candidates.

This system is extremely accurate in measuring the electrons involved in the reaction because they can address quinone directly. Conversely, for the study of any PMP, planar supported lipid membranes can also be constructed on quartz-crystal microbalance where protein-binding events (i.e., between MMPs and MAPs) can be easily monitored [55,57,60]. In these systems the proteins can be investigated in their native environment, which is highly important when testing the reaction of the enzyme to possible drugs. By closely analyzing their function and structure, a molecular framework can be built, on which the action of candidate inhibitors or activators can be predicted. 

Ultimately, new insights into the family of peripheral membrane proteins can lead to ground-breaking advances in the treatment of different severe diseases, from Alzheimer’s to African sleeping sickness.

## Figures and Tables

**Table 1:** Examples of PMPs as key drug targets. The examples of pathogenic organisms in which the protein exists, the effect of the corresponding disease on human health, the currently available drug targets, and the homologues among different domains are described for type-II NADH dehydrogenase [24,25,26,27,28,29], periplasmic nitrate reductase [30,31,32,33], CymA, alkaline phosphatase [34,35], ecto-5′-nucleotidase [36,37], acetylcholine esterase [38,39], alternative oxidase [40,41], cytochrome *c* [42,43], and dihydroorotate dehydrogenase [42,44].

Example Protein (Model Organism)	Examples of Pathogenic Organism(s)	Effect on Human Health	Current Available Drugs/Treatments	Homologues among Domains/Species
Type-II NADH dehydrogenase (NDH-2) (*Caldalkalibacillus thermarum*)	*Mycobacterium tuberculosis*	Tuberculosis; 1.4M deaths worldwide in 2019 [24].	Bedaquiline (against *M. tuberculosis*)NDH-2 targeting thioquinazoline (TQZ)-based and tetrahydroindazole (THI)-based inhibitor candidates [25].	Not reported in mammalian biology; is in prokaryotes and yeast.
	*Staphylococcus aureus*	Opportunistic and nosocomial infections, 50,000 deaths/year in the USA [26].		
	*Escherichia coli*	Gastrointestinal infections causing an estimated 325,000 deaths in developing countries [27]. Cause of 90% of urinary tract infections, 135M cases/year [28]. Horizontal gene transfer of antibiotic resistance to other species [29].		
Periplasmic nitrate reductase (Nap) (*Cupriavidus necator*, *Rhodobacter sphaeroides*)	*Haemophilus influenzae*	Respiratory disease; 199,000 deaths of children/year in 2008 [30].	Not reported targeting Nap. Cefotaxime 80% effective against extensive drug resistant (XDR) strains [31].	Reported in prokaryotes (specifically in bacteria) and eukaryotes; also, in humans.
	*Pseudomonas aeruginosa*	Sixth most common nosocomial pathogen in the USA [32]. Lung infection; 2700 deaths/year in the USA [33].	Not reported targeting Nap. Against multi-drug-resistant strains, cefiderocol and imipenem-cilastatin/relebactam in phase II clinical trials [32].	
CymA	*Shewanella putrificans* *Shewanella alga*	Food spoilage, necrosis, seafood toxin producing (opportunistic pathogen).	N/A	Reported in prokaryotes (specifically in bacteria). Not reported in mammalian biology.
Alkaline phosphatase (AP) (*Homo sapiens*)	Causes disease in humans	Hydroxyapatite deposition disease (HADD) [34].	Paracetamol and/or nonsteroidal anti-inflammatory drugs, barbotage, and steroid injections for severe cases [35].	Reported in prokaryotes (specifically in bacteria) and eukaryotes; also, in humans.
Ecto-5′-nucleotidase (CD73) (*Homo sapiens*)	N/A	Tumor progression; 47,050 deaths/year in the USA in 2020 [36].	Monoclonal antibodies: CPI-006, CPI-444, oleclumab, TJ004309, NZV930, and BMS-986179 [37].	Reported in prokaryotes (specifically in bacteria) and eukaryotes; also, in humans.
Acetylcholine esterase (*Homo sapiens*)	Causes disease in humans	Senile plaque formation (Alzheimer’s disease); 122,019 deaths/year in the USA in 2018 [38].	Donepezil, rivastigmine (Exelon) and galantamine (Razadyne, Nivalin) [39].	Reported in prokaryotes (specifically in bacteria) and eukaryotes; also, in humans.
Alternative oxidase (AO) (*Trypanosoma brucei*)	*Trypanosoma brucei*	African trypanosomiasis (sleeping sickness); 116 deaths in 2019 [40].	Pentamidine (early stage), nifurtimox and eflornithine (late stage) for *T. brucei gambiense*; Suramin (early stage) and melarsoprol (late stage) for *T. brucei rhodesiense* [41].	Reported in prokaryotes (specifically in bacteria) and eukaryotes. Not reported in mammalian biology.
Cytochrome *c*	N/A	Inhibits cancer progression;9,900,000 total cancer deaths/year [42].	Cisplatin [43].	Reported in prokaryotes (specifically in bacteria) and eukaryotes; also, in humans.
Dihydroorotate dehydrogenase (DHODH) (*Homo sapiens*)	N/A	Inhibits cancer progression; 9,900,000 total cancer deaths/year [42].	Brequinar and leflunomide [44].	Reported in prokaryotes (specifically in bacteria) and eukaryotes; also, in humans.
